# Preparing for the implementation of anti-amyloid therapies in Europe: Assessing real-world eligibility for lecanemab and donanemab in a Swedish memory clinic

**DOI:** 10.1016/j.tjpad.2025.100476

**Published:** 2026-01-15

**Authors:** Anna Rosenberg, Alina Solomon, Alexandre Bonnard, Makrina Daniilidou, Göran Hagman, Anette Hall, Anna Matton, Ulf Öhlund-Wistbacka, Eric Westman, Miia Kivipelto

**Affiliations:** a Department of Neurobiology, Care Sciences and Society, Karolinska Institutet, Stockholm, Sweden; b Department of Public Health, Lifestyles and Living Environments, Finnish Institute for Health and Welfare, Helsinki, Finland; c Department of Neurology, Institute of Clinical Medicine, University of Eastern Finland, Kuopio, Finland; d Ageing Epidemiology (AGE) Research Unit, School of Public Health, Imperial College London, London, UK; e Theme Inflammation and Aging, Karolinska University Hospital, Stockholm, Sweden; f FINGERS Brain Health Institute, Stockholm, Sweden; g Institute of Public Health and Clinical Nutrition, University of Eastern Finland, Kuopio, Finland

**Keywords:** Alzheimer's disease, Anti-amyloid antibody, Lecanemab, Donanemab, Eligibility, Real world evidence, Memory clinic

## Abstract

Lecanemab and donanemab are the first anti-Aβ treatments to receive approval in Europe. Eligibility criteria are strict, eg., *APOE* ε4/4 carriers are excluded. Successful implementation in public healthcare hinges on accurate estimates of eligibility rates in settings which will be the first to roll out the treatments (specialized memory clinics with early disease stages). We applied the appropriate use recommendations (AUR) to assess treatment eligibility in a Swedish tertiary memory clinic where Aβ and *APOE* assessments are routinely performed. Of the full cohort (*N* = 410), 26 and 25 patients met the AUR criteria for lecanemab and donanemab, respectively (6 %; partial overlap between the groups). After excluding *APOE* ε4/4 carriers in line with the European guidelines, only 14 and 13 patients remained eligible (3 %). In clinics with younger populations, a significant percentage of potentially eligible patients are likely to have the *APOE* ε4/4 genotype. These findings are important to inform the implementation of anti-Aβ treatments.

## 1 Introduction

The recent introduction of the first disease-modifying therapies (DMTs), the anti-amyloid-β (Aβ) monoclonal antibodies, has transformed the Alzheimer’s disease (AD) therapeutic landscape. Two such therapies are currently available: lecanemab (Leqembi®) and donanemab (Kisunla®). The commercial development of a third agent, aducanumab (Aduhelm®), was discontinued in 2024. Aducanumab and donanemab primarily target insoluble Aβ plaques, whereas lecanemab is designed to bind the soluble forms of Aβ (protofibrils).

In April 2025, lecanemab became the first anti-Aβ therapy to receive marketing authorization from the European Commission [1,2], and in September 2025, donanemab was approved [3,4]. In the European Union, lecanemab and donanemab are indicated for patients with mild cognitive impairment (MCI) or mild dementia due to AD, provided that Aβ pathology is confirmed, and no medical exclusion criteria are present. Exclusion criteria include e.g. the use of anticoagulation therapy and apolipoprotein E (*APOE*) ε4/4 genotype which increase the risk of amyloid-related imaging abnormalities (ARIA) [5]. To support the safe and effective use of anti-Aβ therapies like lecanemab and donanemab in clinical practice, expert groups have published appropriate use recommendations (AUR) [[6], [7], [8], [9]]. These guidelines provide detailed recommendations on patient selection, monitoring protocols, adverse event management, and patient communication strategies, with the goal of facilitating real-world implementation of anti-Aβ therapies. In addition to the AURs, the first real-world experiences with anti-Aβ therapies outside Europe are beginning to be reported, providing valuable insights into the patient populations, therapeutic benefits, and safety aspects in routine clinical practice [10,11].

A critical step in the successful implementation of anti-Aβ therapies in public healthcare is understanding how many patients in clinical settings could be eligible for treatment. Previous studies have assessed real-world eligibility for anti-Aβ therapies in different settings [[12], [13], [14], [15], [16], [17], [18], [19], [20], [21], [22], [23], [24]]. Our earlier work was among the first to estimate eligibility in a memory clinic population, using the only AUR criteria available at the time (AUR for aducanumab) [12]. The study was conducted in a setting where the new DMTs are likely to be first rolled out once available in Europe (tertiary memory clinics with predominantly early disease stages). The present study, conducted at the same memory clinic, aimed to estimate eligibility for lecanemab and donanemab. Eligibility was assessed by applying the AUR criteria and additionally considering the *APOE*-related exclusion criteria, in line with the European treatment guidelines.

## 2 METHODS

### 2.1 Study setting and participants

The study was conducted at the Karolinska University Hospital Medical Unit Aging Memory clinic in Solna, Stockholm, Sweden. This outpatient clinic serves individuals with cognitive concerns from the northern Stockholm catchment area, as well as patients under the age of 70 from across the Stockholm region. Referrals are typically made by general practitioners in primary or occupational health care.

The clinic is an example of a setting that is already equipped with the specialized personnel and diagnostic infrastructure necessary for the implementation of new DMTs for AD. The diagnostic protocol, described in detail in a previous publication [12], includes a comprehensive medical and neurological examination, medical and informant-based history, neuropsychological evaluation, blood tests, cerebrospinal fluid (CSF) biomarker assessment, *APOE* genotyping, and magnetic resonance imaging (MRI). MRI evaluation includes both visual inspection and automated analysis of regional brain volumes, white matter hyperintensities, and dementia-related scales (e.g., medial temporal atrophy, MTA) using cNeuro® cMRI software [25]. Additional investigations are performed as needed. A consensus diagnosis is established based on all available clinical and biomarker data.

All patients undergo the full diagnostic workup, including AD-related biomarker testing, regardless of the suspected etiology of cognitive symptoms. Patients are routinely invited to provide informed consent for inclusion in the Karolinska University Hospital clinical research database and biobank (ethical approvals Dnr 2011/1987-31/4, 2020-06484).

In line with our previous work [12], we included in this study all consecutive patients who had their first diagnostic visit between April 2018 and February 2021, provided informed consent for research, and had complete clinical, imaging, and CSF biomarker data (*N* = 410).

### 2.2 Eligibility for lecanemab and donanemab treatment

To estimate the number of patients potentially eligible for anti-Aβ treatment, we applied the inclusion and exclusion criteria outlined in the appropriate use recommendations (AUR) for lecanemab [8] and donanemab [9]. We also incorporated eligibility criteria defined by EMA in the label guidelines [5].

Eligibility was assessed using a stepwise approach. Supplementary Tables 1 and 2 detail the AUR criteria and their operationalization in this study. Briefly, patients were considered eligible if they met the following conditions:

1) Confirmed Aβ pathology, defined as abnormal CSF Aβ42 based on standard laboratory cut-offs used in routine clinical practice (for Innotest, used until 08/21/2019, ≤550 pg/mL; for Lumipulse used thereafter, ≤599 pg/mL). In an additional analysis, we applied the more lenient, data-driven cut-off determined in our previous study in this cohort (<707 pg/mL, all samples) [12]. While the AURs recommend both abnormal phosphorylated tau and Aβ42 for CSF-based confirmation, we followed the EMA’s definition in the label guidelines, which requires evidence of Aβ pathology alone.
2) Diagnosis of AD-type dementia or MCI, coded as ICD-10 F00, G30, or F06.7, with no evidence of a non-AD neurological disorder.
3) Age and cognitive criteria, excluding patients <50 or >90 years (lecanemab) or <60 or >85 years (donanemab), and those with substantial cognitive impairment, i.e., Mini-Mental State Examination (MMSE) <22 or Montreal Cognitive Assessment (MoCA) <17 (lecanemab), or MMSE <20 or MoCA<13 (donanemab).
4) No apparent medical exclusion criteria, including e.g., use of anticoagulants. *APOE* genotyping was also required.

In addition, to obtain the final eligibility rates, *APOE* genotype was considered and patients with ε4/4 genotype were excluded in line with the European treatment guidelines, due to an elevated risk of ARIA [5].

Full MRI reports were not available to assess all imaging-related contraindications (e.g., macro- or microhaemorrhages), and it was not possible to evaluate all medical exclusions and medical history/records individually. The AURs specify that comorbid conditions should be stable or well-managed, and in line with this, the clinic’s referral process requires stabilization of major medical or psychiatric conditions (e.g., cardiovascular disease, depression, cancer) prior to examination at the clinic.

## 3 Results

### 3.1 Patient characteristics

A total of 410 memory clinic patients with clinical, CSF, and imaging biomarker data were included in the analysis. The mean age was 59 years (SD ± 7), and 56 % were women. Twenty-five percent of the patients (*N* = 102) had a clinical diagnosis of dementia (67 % of the cases AD-type); 23 % (*N* = 94) had MCI, and 52 % (*N* = 214) had subjective cognitive impairment (SCI)/other diagnosis. Among the 399 patients with *APOE* genotyping, 40 % were ε4 carriers (8 % ε4/4). Of patients with abnormal CSF Aβ42, 63 % were ε4 carriers (31 % ε4/4). Detailed cohort characteristics have been reported previously [12].

### 3.2 Eligibility for lecanemab and donanemab treatment

**Fig. 1:**
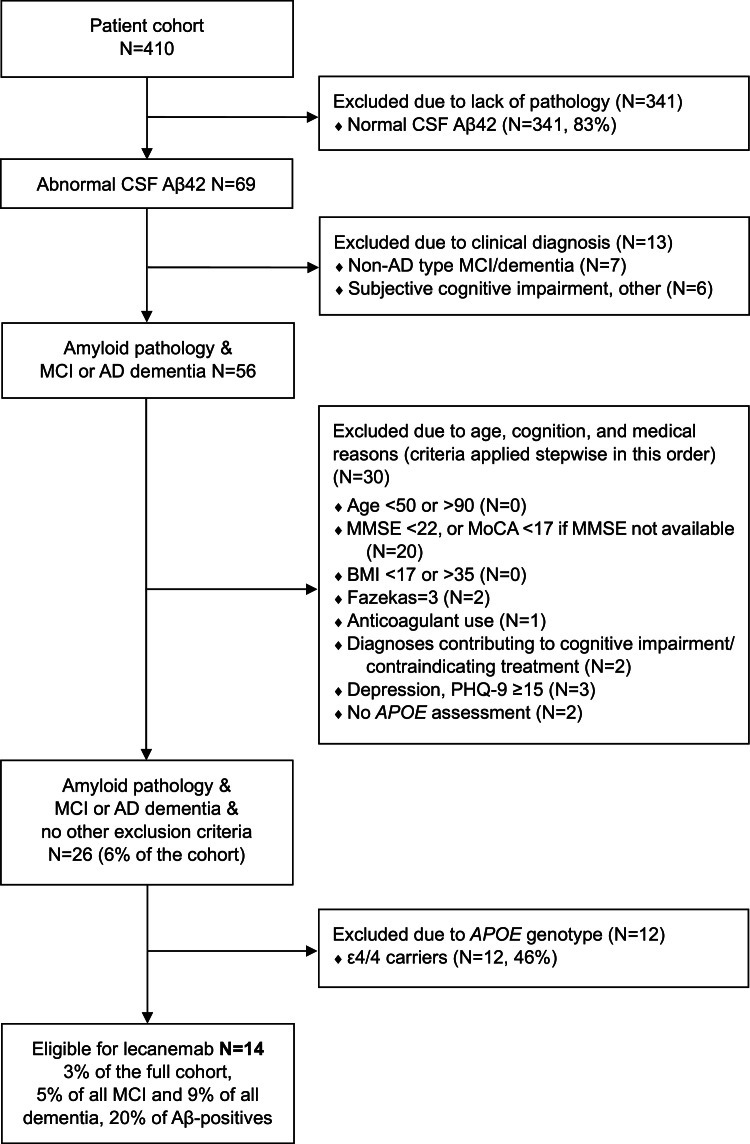
Potential eligibility for lecanemab treatment. Patient eligibility was assessed according to the lecanemab appropriate use recommendations (AUR) (Cummings et al., 2023). Abnormal CSF Aβ42 (based on laboratory cut-offs used in routine clinical practice), regardless of tau, was considered sufficient evidence for AD pathology. Exclusion of APOE ε4/4 carriers (final step) is the EMA requirement for patients in the EU.

**Fig. 2:**
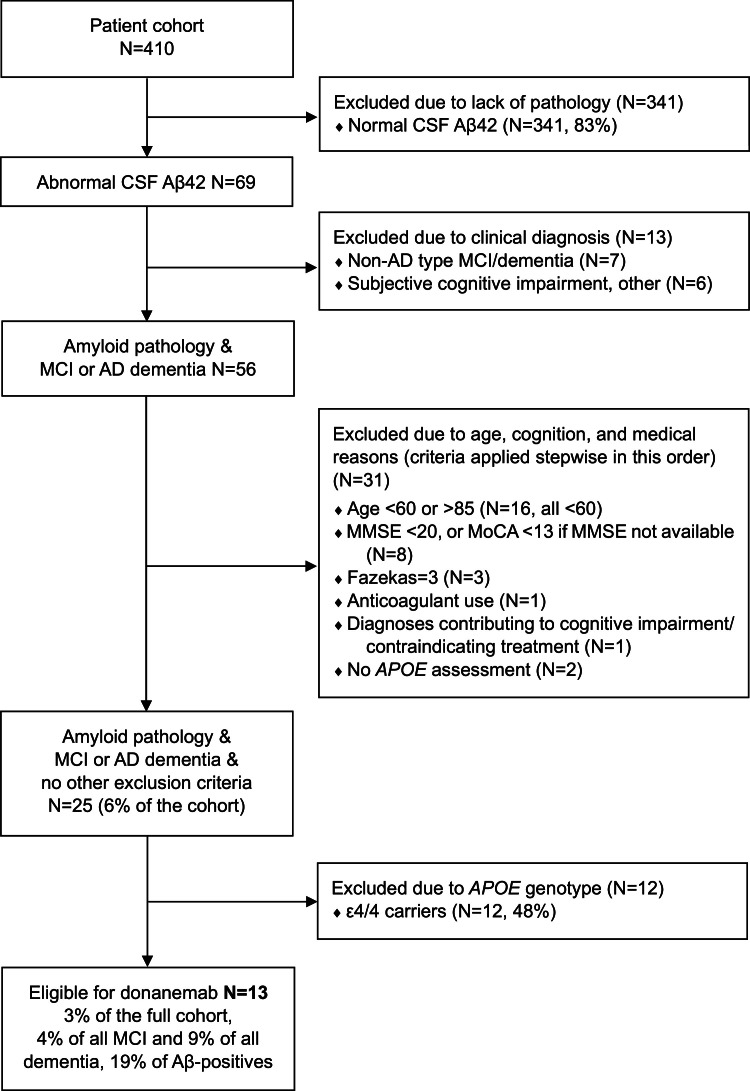
Potential eligibility for donanemab treatment. Patient eligibility was assessed according to the donanemab appropriate use recommendations (AUR) (Rabinovici et al., 2025). Abnormal CSF Aβ42 (based on laboratory cut-offs used in routine clinical practice), regardless of tau, was considered sufficient evidence for AD pathology. Exclusion of APOE ε4/4 carriers (final step) is the EMA requirement for patients in the EU.

Of the 410 patients, 341 (83 %) were excluded due to lack of Aβ pathology, defined as normal CSF Aβ42 (based on the standard laboratory cut-offs used in clinical practice). Among the remaining 69 patients with confirmed Aβ pathology, 13 were excluded based on clinical diagnosis (non-AD MCI/ dementia, *N* = 7; SCI/other, *N* = 6). Criteria related to age, global cognition, medical reasons, and *APOE* availability excluded further patients. A detailed breakdown of the eligibility analysis and number of patients excluded at each step is provided in Fig. 1, Fig. 2.

Without considering the *APOE* genotype, 26 patients met the AUR eligibility criteria for lecanemab (6 % of the full cohort; 12 % of all MCI and 15 % of all dementia patients; 38 % of Aβ-positives). Twenty-five patients met the AUR eligibility criteria for donanemab (6 % of the full cohort; 11 % of all MCI and 15 % of all dementia patients; 36 % of Aβ-positives). Groups partially overlapped (*N* = 20 eligible for both, *N* = 6 only for lecanemab, *N* = 5 only for donanemab).

Patients potentially eligible for lecanemab were on average 62.7 years old (SD ± 4.8) and 14 of 26 (54 %) were women. Fifteen patients (58 %) had a clinical diagnosis of dementia (MCI *N* = 11, 42 %). Mean MMSE was 26.9 (SD ± 2.5), and MoCA 21.3 (SD ± 4.2). Median MTA (visual rating) was 1 (IQR 0.5–1.5). Eight patients (31 %) had the *APOE* ε3/4 genotype, and 12 patients (46 %) had the ε4/4 genotype. In terms of biomarker profile (ATN; defined based on CSF Aβ42, CSF p-tau181, and MTA on MRI), 10 patients were *A*+*T*+*N*+, 9 were *A*+*T*+*N*−, 5 were *A*+*T*−*N*−, and 2 were *A*+*T*−*N*+.

Patients potentially eligible for donanemab were slightly older on average due to the stricter age criteria (mean age 64.2 ± 3.2 years) and 16 of 25 (64 %) were women. Fifteen patients (60 %) had a clinical diagnosis of dementia (MCI *N* = 10, 40 %). Mean MMSE was 25.7 (SD ± 3.2), and MoCA 20.4 (SD ± 4.2), reflecting the more lenient criteria for global cognition. Median MTA (visual rating) was 1.125 (IQR 0.5–2). Nine patients (36 %) had the *APOE* ε3/4 genotype, and 12 patients (48 %) had the ε4/4 genotype. In terms of biomarker profile, 9 patients were *A*+*T*+*N*+, 9 were *A*+*T*+*N*−, 4 were *A*+*T*−*N*−, and 3 were *A*+*T*−*N*+.

After considering the *APOE* genotype and excluding ε4/4 carriers, 14 patients remained potentially eligible for lecanemab (Fig. 1; 3 % of the full cohort; 5 % of all MCI and 9 % of all dementia patients; 20 % of Aβ-positives). Thirteen patients remained potentially eligible for donanemab (Fig. 2; 3 % of the full cohort; 4 % of all MCI and 9 % of all dementia patients; 19 % of Aβ-positives).

In an additional analysis, we applied the more lenient data-driven cut-off for CSF Aβ42 to determine amyloid positivity and repeated the eligibility analysis (Supplementary Figure 1 and 2). Without considering the *APOE* genotype, 47 patients met the AUR eligibility criteria for lecanemab (11 % of the full cohort; 19 % of all MCI and 28 % of all dementia patients; 41 % of Aβ-positives). Forty-three patients met the AUR eligibility criteria for donanemab (10 % of the full cohort; 18 % of all MCI and 25 % of all dementia patients; 37 % of Aβ-positives). Groups partially overlapped (*N* = 36 eligible for both, *N* = 11 only for lecanemab, *N* = 7 only for donanemab). After considering the *APOE* genotype and excluding ε4/4 carriers, 29 patients remained potentially eligible for lecanemab (7 % of the full cohort; 11 % of all MCI and 19 % of all dementia patients; 25 % of Aβ-positives). Twenty-seven patients remained potentially eligible for donanemab (7 % of the full cohort; 11 % of all MCI and 17 % of all dementia patients; 23 % of Aβ-positives).

## 4 Discussion

We assessed real-world eligibility for anti-Aβ therapies donanemab and lecanemab in a Swedish university hospital tertiary memory clinic where CSF, neuroimaging, and *APOE* genotyping are routinely included in the diagnostic workup. This setting is representative of highly specialized clinics which are already equipped with the infrastructure required for implementing the new AD DMTs. Given its profile (focus on early disease/predementia stages and responsibility for younger patients), this clinic also exemplifies a setting that is well-positioned to roll out the new anti-Aβ therapies to reach the optimal patient group. We found that 6 % of the patients met the AUR eligibility criteria for donanemab and lecanemab. With *APOE* ε4/4 being the most common genotype among these patients, the final proportion of eligible patients after excluding ε4/4 carriers (in line with the European treatment guidelines) was 3 %. Applying more lenient data-driven cut-offs for CSF Aβ42 to determine amyloid positivity increased the eligibility rate but it remained low (7 %, after excluding ε4/4 carriers).

Our findings align with previous real-world studies, which have consistently reported relatively low eligibility rates for anti-Aβ therapies [[12], [13], [14], [15], [16], [17], [18], [19], [20], [21], [22], [23], [24]]. Estimates vary depending on the clinical setting (memory clinic vs. population-based), the antibody evaluated (e.g., aducanumab vs. lecanemab), and how eligibility criteria are chosen and operationalized (trial-based vs. AUR or label guidelines). In our previous study at the same clinic, 7 % of patients were eligible for aducanumab using its AUR [12]. The choice of biomarkers and their cut-offs had some effect on these estimates; in line with the current study, applying more lenient, data-driven cut-offs for biomarkers increased the proportions of eligible patients. Eligibility rates of ∼10 % have been reported in Italian memory clinics [16,18], while lower rates were observed in Austria (2 %) and Ireland (6 %), likely because most patients lacked an Aβ assessment [15,19]. In a recent Dutch memory clinic study, 6 % of patients met the treatment criteria for lecanemab [24]. Similarly to our clinic, this patient population was relatively young (mean age 63 years), and the standard diagnostic workflow included biomarker assessments. Community-based studies have also reported limited eligibility. In the Swedish Gothenburg H70 cohort, 6 % met the lecanemab inclusion criteria [17], while in the Rotterdam Study, 8–15 % were eligible for aducanumab, lecanemab, and donanemab (Aβ status was algorithmically predicted) [23]. In the Mayo Clinic Study of Aging, 5–8 % of Aβ-positive individuals with MCI or mild dementia were eligible for aducanumab and lecanemab [14]. In this study, comorbidities and imaging findings were common exclusion criteria.

An important observation in our study is the high proportion of *APOE* ε4/4 carriers among those initially eligible for anti-Aβ therapies, leading to further exclusions as per the European treatment guidelines. Altogether, 46 % and 48 % of those otherwise potentially eligible for lecanemab and donanemab, respectively, were excluded due to *APOE* (38 % and 37 % when a more lenient data-driven cut-off was applied to determine amyloid positivity). Overall, 8 % of all patients in our cohort were *APOE* ε4/4 carriers (31 % of those with abnormal CSF Aβ42). A recent Dutch memory clinic study found that 26 % of those otherwise eligible for lecanemab were ε4/4 carriers [24]; the Mayo Clinic study noted that 74 % of eligible individuals were ε4 carriers (proportion of ε4/4 was not reported) [14]. The overrepresentation of ε4/4 carriers may not be that surprising given the strong association between *APOE* ε4 and AD pathology [26], but it could be particularly evident in highly specialized tertiary memory clinics like ours that include younger patients (including early-onset and potentially also familial cases; the Karolinska University Hospital Solna unit serves patients under the age of 70 from across the Stockholm region). Our results show that in real-world specialized memory clinics where anti-Aβ therapies are most likely to be first prescribed, the proportion of ε4/4 carriers could be higher than previously estimated based on clinical trial data (in CLARITY-AD, 16 % of treated participants were ε4/4 carriers [5,27]). This is important to consider when planning the implementation of anti-Aβ therapies.

Our study has some limitations. We could not assess all medical and safety-related exclusion criteria, such as those documented in detailed medical records. This may slightly overestimate eligibility. However, our clinic’s referral process requires stabilization of major medical conditions prior to assessment, which likely mitigates this effect. Lack of access to full MRI reports can also slightly overestimate eligibility, although fewer imaging-related exclusions are expected in our patient cohort than in older cohorts. Still, imaging-related contraindications to anti-Aβ treatment occur even in younger memory clinic patient populations [24]. Finally, our cohort is relatively young (reflecting the Swedish diagnostic pathway in which dementia cases are often managed in primary care), and therefore not representative of patient populations in many other memory clinics. In older and community-based populations, comorbidities and imaging findings may substantially reduce eligibility [14]. Nevertheless, our study provides realistic estimates of anti-Aβ treatment eligibility in the type of settings where DMTs like lecanemab and donanemab are most likely to be rolled out for optimal benefit-risk ratio (highly specialized settings with patients predominantly at earlier, predementia disease stages). In a recent US study reporting real-world experiences with lecanemab treatment, the risk of developing symptomatic ARIA was considerably higher in patients with mild dementia (CDR 1) than in patients with MCI-level impairment (CDR 0.5), underlining the importance of early intervention at milder disease stages [11].

Real-world eligibility studies such as ours are essential to guide the clinical rollout of lecanemab and donanemab as they inform implementation strategies and resource planning. Anti-Aβ therapies represent a breakthrough in the field, but most patients with cognitive impairment may not qualify due to lack of required brain pathology, or increased risk of serious side effects (when applying the current recommendations). In addition to the few already published reports, future real-world analyses of lecanemab- and donanemab-treated patients will be important to identify subgroups most likely to benefit or experience harm, and to refine eligibility criteria accordingly. Studies in different populations are needed as the risk profiles may vary. Furthermore, the potential role of co-pathologies in modulating treatment response and risk profile warrants investigation, as pathologies other than Aβ are common among those eligible for anti-Aβ therapies [22]. Given the heterogeneity of AD, and the limited real-world eligibility for anti-Aβ therapies, it remains important to investigate DMTs with alternative mechanisms of action, as well as different combination approaches (e.g., anti-Aβ combined with other pharmacological or non-pharmacological interventions).

## Abbreviations

AD: Alzheimer's Disease

Aβ: Amyloid-β

APOE: Apolipoprotein E

ARIA: Amyloid-Related Imaging Abnormalities

ATN: Amyloid, Tau, Neurodegeneration (biomarker classification)

AUR: Appropriate Use Recommendations

CSF: Cerebrospinal Fluid

DMT: Disease-Modifying Therapy

EMA: European Medicines Agency

MCI: Mild Cognitive Impairment

MMSE: Mini-Mental State Examination

MoCA: Montreal Cognitive Assessment

MRI: Magnetic Resonance Imaging

MTA: Medial Temporal Atrophy

SCI: Subjective Cognitive Impairment

## Funding

This project was supported by funding from the Innovative Health Initiative Joint Undertaking (JU) -PROMINENT under grant agreement No. 101112145, with support from BioArctic for this project. MK received funding from the Innovative Health Initiative (IHI) Joint Undertaking (JU) -PROMINENT under grant agreement No. 101112145; EU Innovative Health Initiative Joint Undertaking (IHI JU) AD-RIDDLE under grant agreement No. 101132933; Alzheimer’s Drug Discovery Foundation (USA); Region Stockholm research grant (ALF, Sweden); Center for Innovative Medicine (CIMED) at Karolinska Institute (Sweden); Stiftelsen Stockholms sjukhem (Sweden); Swedish research council for health, working life and welfare (FORTE). AS received funding from the EU Joint Programme - Neurodegenerative Disease Research (JPND) Multi-MeMo grant (Academy of Finland No. 357810); Innovative Health Initiative (IHI) Joint Undertaking (JU) -PROMINENT under grant agreement No. 101112145; EU Innovative Health Initiative Joint Undertaking (IHI JU) AD-RIDDLE, under grant agreement No. 101132933; Alzheimerfonden (Sweden); Juho Vainio Foundation (Finland); Finnish Cultural Foundation (Finland); Yrjö Jahnsson Foundation (Finland). EW received funding from the Swedish Research Council, Center for Innovative Medicine (CIMED) at Karolinska Institute (Sweden), the regional agreement on medical training and clinical research (ALF) between Stockholm County Council and Karolinska Institutet (Sweden), The Swedish Brain Foundation, The Swedish Alzheimer's Foundation (Alzheimerfonden) No. AF-967495, AF-980387, The Swedish Parkinson's foundation, EU Innovative Health Initiative Joint Undertaking (IHI JU) AD-RIDDLE; King Gustaf V:s and Queen Victorias Foundation (Sweden), The Swedish Dementia Foundation, Olle Engkvists Foundation (Sweden) as well as Birgitta and Sten Westerberg for additional financial support. The sponsors had no role in the design and conduct of the study; in the collection, analysis, and interpretation of data; in the preparation of the manuscript; or in the review or approval of the manuscript.

## Acknowledgements

Maria Ellgren, Mats Ekelund and Sven Eriksson (BioArctic) are acknowledged for their input on data interpretation.

## Data availability

Professor Kivipelto's research team is open to requests for data collected in this study. Study plan (including the research question, planned analysis, and data required) will be evaluated on a case-by-case basis. Shared data will encompass the data dictionary and de-identified data only. Analysis will be conducted in collaboration with Professor Kivipelto's team. Access is subject to the GEDOC legal framework. An access agreement will be prepared.

## Declaration of the use of generative AI and AI-assisted technologies

No generative AI or AI-assisted technologies were used in the writing process or in the figures of this manuscript.

## CRediT authorship contribution statement

**Anna Rosenberg:** Writing – review & editing, Writing – original draft, Visualization, Validation, Project administration, Methodology, Investigation, Formal analysis, Data curation, Conceptualization. **Alina Solomon:** Writing – review & editing, Supervision, Methodology, Funding acquisition, Conceptualization. **Alexandre Bonnard:** Writing – review & editing, Resources, Investigation. **Makrina Daniilidou:** Writing – review & editing, Project administration, Methodology. **Göran Hagman:** Writing – review & editing, Resources, Investigation, Data curation. **Anette Hall:** Writing – review & editing, Software, Methodology, Formal analysis. **Anna Matton:** Writing – review & editing, Resources, Project administration, Methodology, Funding acquisition. **Ulf Öhlund-Wistbacka:** Writing – review & editing, Resources, Methodology, Investigation, Data curation. **Eric Westman:** Writing – review & editing, Supervision, Funding acquisition. **Miia Kivipelto:** Writing – review & editing, Supervision, Resources, Project administration, Funding acquisition, Conceptualization.

## Declaration of interests

The authors declare the following financial interests/personal relationships which may be considered as potential competing interests: Miia Kivipelto reports a relationship with BioArctic AB that includes: scientific advisory board membership. Miia Kivipelto reports a relationship with Eisai Inc that includes: scientific advisory board membership. Miia Kivipelto reports a relationship with Eli Lilly and Company that includes: scientific advisory board membership. Miia Kivipelto reports a relationship with Nestle that includes: scientific advisory board membership. Miia Kivipelto reports a relationship with Combinostics Ltd that includes: scientific advisory board membership. If there are other authors, they declare that they have no known competing financial interests or personal relationships that could have appeared to influence the work reported in this paper.

## Appendix Supplementary materials

Image, application 1
