# Supplementary material for: Preparing for the implementation of anti-amyloid therapies in Europe: Assessing real-world eligibility for lecanemab and donanemab in a Swedish memory clinic
Source: J Prev Alzheimers Dis. 2026 Jan 15;13(3):100476. doi: 10.1016/j.tjpad.2025.100476 (PMC12835597; doi:10.1016/j.tjpad.2025.100476)
Supplement: Supplementary file 1 [file mmc1.docx]

**SUPPLEMENTARY MATERIAL**

Supplementary Table 1. The Appropriate Use Recommendations (AUR) patient eligibility criteria for lecanemab and their operationalization in this study

| **Eligibility criteria** | **Operationalization in this study** |
| --- | --- |
| **Inclusion criteria** | |
| Presence of amyloid pathology:  Positive amyloid PET or CSF indicative of AD | Abnormal CSF Aβ42, based on the cut-off provided by the laboratory/manufacturer and routinely used in clinical practice (main analysis), or data-driven cut-offs determined in our previous study, Rosenberg et al., 2022 (additional analysis)* |
| Diagnosis: clinical diagnosis of MCI or  mild AD dementia | Clinical diagnosis of MCI (ICD-10 F06.7; no evidence of a non-AD neurological disorder) or AD dementia (ICD-10 F00, G30; including mixed/ atypical) |
| Age: Physician judgement used outside the 50–90-y. range | Age 50–90 years |
| Global cognition: MMSE 22-30 or other screening instrument with a score compatible with early AD | MMSE 22–30, or MoCA 17–30 when MMSE not available (as in our previous study, Rosenberg et al., 2022) |
| BMI: Physician judgement used at extreme BMIs (RCT criteria: BMI >17 and <35) | BMI >17 and <35 kg/m^2^; those with missing BMI information were not excluded. |
| Patients may be on cognitive enhancing agents (donepezil, rivastigmine, galanta­mine, or memantine) for AD. but not on aducanumab | No concomitant AD medication use in this cohort at the time of data collection/ analysis. No impact on eligibility analysis. |
| Patients may be on standard of care for other medical illnesses (see below for specifics regarding anticoagulation) | Participants with or without standard care for other medical conditions were included (see below regarding anticoagulation) |
| Have a care partner or family member(s) who can ensure that the patient has the support needed to be treated with lecanemab | NA |
| Patients, care partners, and appropriate family members should understand the requirements for lecanemab therapy and the potential benefit and potential harm of treatment | NA |
| *APOE* genotyping of all treatment candidates is recommended before initiating lecanemab therapy | *APOE* genotyping is part of the diagnostic routine at this clinic. Patients with no *APOE* assessment were excluded. |
| **Exclusion criteria** | |
| Any medical, neurologic, or psychiatric condition that may be contributing to the cognitive impairment or any non-AD MCI or dementia | Patients with diagnoses other than MCI or AD dementia were excluded, as well as those with diagnoses (ICD-10 codes) contributing to cognitive impairment/ contraindicating treatment. |
| Recent history (within 12 months) of stroke or transient ischemic attacks or any history of seizures  Mental illness (e.g, psychosis), or major depression that interferes with comprehension of the requirements, potential benefit, and potential harms of treatment and are considered by the physician to render the patient unable to comply with management requirements.  Any history of immunologic disease (e.g., lupus erythematosus, rheumatoid arthritis, Crohn’s disease) or systemic treatment with immunosuppressants, immunoglobulins, or monoclonal antibodies or their derivatives  Patients with a bleeding disorder that is not under adequate control.  Unstable medical conditions that may affect or be affected by lecanemab therapy. | Detailed medical history and medical records were not available to fully assess these exclusion criteria.  The standard referral process requires all major illnesses (e.g., cardiovascular, psychiatric, depression, and cancer) and related treatments to be stabilized before referral to our clinic.  Depression: patients with a Patient Health Questionnaire-9 (PHQ-9) score ≥15 were excluded (indicating moderately severe or severe depression) |
| Patients on anticoagulants should not receive lecanemab; tPA should not be administered to individuals on lecanemab.  Use of concomitant aspirin and other antiplatelet therapy permitted (EMA guidelines in Europe). | Patients with anticoagulant treatment were excluded (ATC code B01A; platelet antiaggregation agents B01AC allowed) |
| Imaging:   - Inability to undergo MRI due to claustrophobia, pacemaker, defibrillator, or metal implants - MRI evidence of a non-AD dementia - More than 4 microhemorrhages (defined as 10 mm or less at the greatest diameter); a single macrohemorrhage >10 mm at greatest diameter; an area of superficial siderosis; evidence of vasogenic edema; more than 2 lacunar infarcts or stroke involving a major vascular territory; severe subcortical hyperintensities consistent with a Fazekas score of 3; evidence of amyloid beta-related angiitis; cerebral amyloid angiopathy-related inflammation; or other major intracranial pathology that may cause cognitive impairment. | MRI was conducted for all patients in this cohort and considered in the diagnostic process. Patients with other clinical diagnoses than MCI/AD dementia were excluded.  Patients with Fazekas=3 were excluded.    Full radiologist reports were not available to assess all MRI-related contraindications, e.g., micro- and macrohemorrhages. |

Eligibility was assessed according to the lecanemab appropriate use recommendations (Cummings et al. 2023). Abnormal CSF Aβ42 regardless of tau status was considered sufficient evidence for AD pathology, in line with the European guidelines by EMA (confirmed Aβ pathology). Inclusion and exclusion criteria are listed on the left, and the right column shows how the criteria were applied in our data to define eligibility. NA indicates that the item was not considered in the analysis (information not available).

* CSF Aβ42 was analyzed with Innotest and Lumipulse G-series (Fujirebio Europe) assays, with cut-offs of ≤550 pg/ml (Innotest) and ≤599 pg/ml (Lumipulse) (cut-offs used in routine clinical practice). Data-driven cut-off: <707 pg/ml (all samples)

Supplementary Table 2. Appropriate Use Recommendations (AUR) patient eligibility criteria for donanemab and their operationalization in this study

| **Eligibility criteria** | **Operationalization in this study** |
| --- | --- |
| **Inclusion criteria** | |
| Presence of amyloid pathology:  Positive amyloid PET or CSF indicative of AD pathology | Abnormal CSF Aβ42, based on the cut-off provided by the laboratory/manufacturer and routinely used in clinical practice (main analysis), or data-driven cut-offs determined in our previous study, Rosenberg et al., 2022 (additional analysis)* |
| Diagnosis: clinical diagnosis of MCI or dementia with mild functional impairment, with AD as the suspected etiology based on cognitive-behavioral syndrome | Clinical diagnosis of MCI (ICD-10 F06.7; no evidence of a non-AD neurological disorder) or AD dementia (ICD-10 F00, G30; including mixed/ atypical) |
| Age: Physician judgement used for patients outside the 60–85-year age range | Age 60–85 years |
| Global cognition: MMSE 20–30, MoCA 13–30, or other cognitive screening instrument with a score compatible with early AD | MMSE 20–30, or MoCA 13–30 when MMSE not available |
| Patients may be on cognitive enhancing agents for AD (donepezil, rivastigmine, galantamine, memantine), but not on aducanumab or lecanemab | No concomitant AD medication use in this cohort at the time of data collection/ analysis. No impact on eligibility analysis. |
| Patients may be on standard of care for other medical illnesses (see below for specifics regarding anticoagulation) | Participants with or without standard care for other medical conditions were included (see below regarding anticoagulation) |
| Patients should understand that the effect of donanemab on the ability to have children or its effect on the unborn fetus are unknown | NA |
| Have a care partner or family member(s) who can ensure that the patient has the support needed to be treated with donanemab | NA |
| In the opinion of the clinician, have adequate literacy, vision, and hearing for cognitive testing | The routine diagnostic process includes neuropsychological testing for all patients; no impact on eligibility analysis. |
| Treated patients are reliable and are willing to follow study procedures | NA |
| Patients, care partners, and appropriate family members understand the requirements for donanemab therapy and the potential benefits and harms of treatment. Approval by surrogate decision maker with patient assent may be appropriate if patient lacks capacity to make medical decisions independently | NA |
| *APOE* genotyping is performed prior to initiating treatment to assess an individual's risk of ARIA | *APOE* genotyping is part of the diagnostic routine at this clinic. Patients with no *APOE* assessment were excluded. |
| **Exclusion criteria** | |
| Non-AD neurologic condition that may be significantly contributing to cognitive or behavioral impairment | Patients with diagnoses other than MCI or AD dementia were excluded, as well as those with diagnoses (ICD-10 codes) contributing to cognitive impairment/ contraindicating treatment. |
| Autosomal dominant AD, if the mutation is associated with high prevalence and burden of cerebral amyloid angiopathy (CAA) | NA. Genetic testing for familial AD is not routinely conducted, no information about potential autosomal dominant AD cases. |
| AD due to Down syndrome | No patients with Down syndrome in this cohort. No impact on eligibility analysis. |
| Recent history (within 12 months) of stroke or transient ischemic attacks or any history of seizures  Psychiatric disorder, suicidal ideation, or history of alcohol or substance use that interferes with comprehension of the requirements, potential benefit, and potential harms of treatment and are considered by the clinician to render the patient unable to comply with treatment requirements  Medical condition that may significantly contribute to cognitive impairment or interfere with the patients’ ability to participate in treatment or the clinician's ability to assess the patient  Physical condition that impairs the ability of the patient to have intravenous infusions  Patients with known allergies to donanemab or related compounds are excluded  History of immunologic disease (e.g., systemic lupus erythematosus, rheumatoid arthritis, Crohn's disease) or current systemic treatment with immunosuppressants, immunoglobulins, or monoclonal antibodies or their derivatives  Active cancer that interferes with the ability to comply with donanemab treatment  Patients with a bleeding disorder that is not under adequate control.  Findings on physical or neurological examination, vital signs, or laboratory tests that may significantly contribute to cognitive impairment or interfere with the patients’ ability to participate in treatment or the clinician's ability to assess the patient | NA. Detailed medical history and medical records were not available to fully assess these exclusion criteria.  The standard referral process requires all major illnesses (e.g., cardiovascular, psychiatric, depression, and cancer) and related treatments to be stabilized before referral to our clinic. |
| If previously treated with an Aβ-targeting therapy, must allow washout period of >5 half-lives; must demonstrate current biomarker evidence of Aβ plaques, and must not meet other clinical or MRI exclusion criteria | No history of anti-Aβ treatment in this cohort. No impact on eligibility analysis. |
| Patients on anticoagulants (coumadin, dabigatran, rivaroxaban, apixaban, betrixaban, or heparin) should not receive donanemab; thrombolytics should not be administered to individuals on donanemab | Patients with anticoagulant treatment were excluded (ATC code B01A; platelet antiaggregation agents B01AC allowed) |
| Sensitivity or contraindication to amyloid imaging ligands if amyloid PET is required for confirmation of AD | CSF used to determine amyloid pathology in this cohort. No impact on eligibility analysis. |
| Imaging:   - Contraindications for MRI, incl. claustrophobia or the presence of contraindicated metal (ferromagnetic) implants/cardiac pacemaker - Abnormality suggesting a non-AD cause for progressive cognitive impairment or a clinically significant finding that may impact safety - Amyloid-related imaging abnormalities of edema/effusion, more than 4 cerebral micro-hemorrhages, any area of superficial siderosis; any intracerebral hemorrhage greater than 1 cm or severe white matter disease; CAA-related inflammation; evidence of territorial infarcts > 1 cm; more than 2 lacunar infarcts, cerebral contusion, encephalomalacia, brain aneurysms or other vascular malformations, central nervous system infection, and brain tumors, except for small meningiomas or arachnoid cysts. | MRI was conducted for all patients in this cohort and considered in the diagnostic process. Patients with other clinical diagnoses than MCI/AD dementia were excluded.  Patients with Fazekas=3 indicative of severe white matter lesions were excluded.    Full radiologist reports were not available to assess all MRI-related contraindications, e.g., micro- and macrohemorrhages. |

Eligibility was assessed according to the donanemab appropriate use recommendations (Rabinovici et al. 2025). Abnormal CSF Aβ42 regardless of tau status was considered sufficient evidence for AD pathology, like in lecanemab’s case. Inclusion and exclusion criteria are listed on the left, and the right column shows how the criteria were applied in our data to define eligibility. NA indicates that the item was not considered in the analysis (information not available).

* CSF Aβ42 was analyzed with Innotest and Lumipulse G-series (Fujirebio Europe) assays, with cut-offs of ≤550 pg/ml (Innotest) and ≤599 pg/ml (Lumipulse) (cut-offs used in routine clinical practice). Data-driven cut-off: <707 pg/ml (all samples)


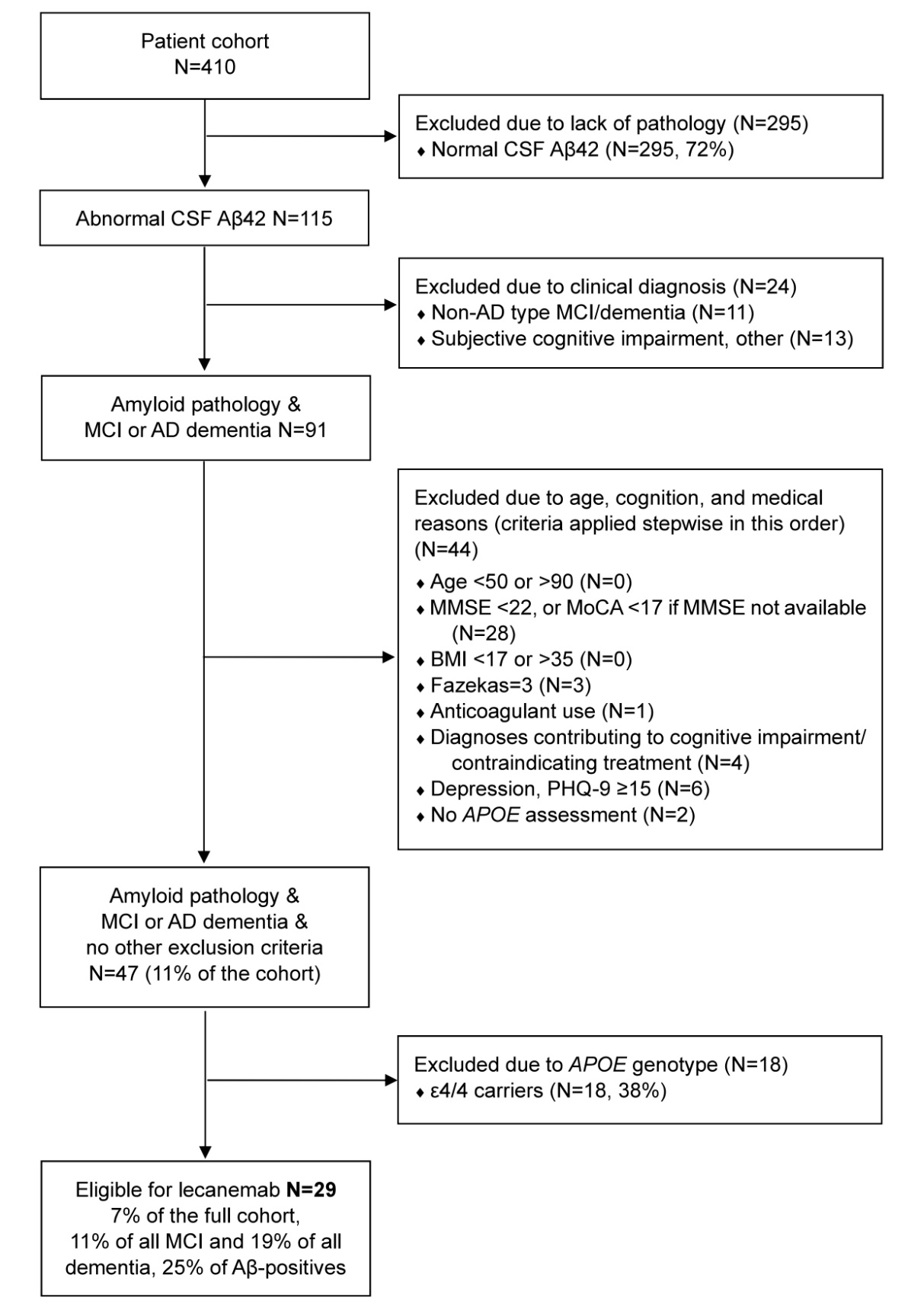


Supplementary Figure 1. Potential eligibility for lecanemab treatment, using the data-driven cut-off for CSF Aβ42 to determine amyloid pathology

Eligibility was assessed according to the lecanemab appropriate use recommendations (AUR) (Cummings et al., 2023). Abnormal CSF Aβ42 regardless of tau was considered sufficient evidence for AD pathology. Exclusion of *APOE* ε4/4 carriers (final step) is the EMA requirement for patients in the EU.


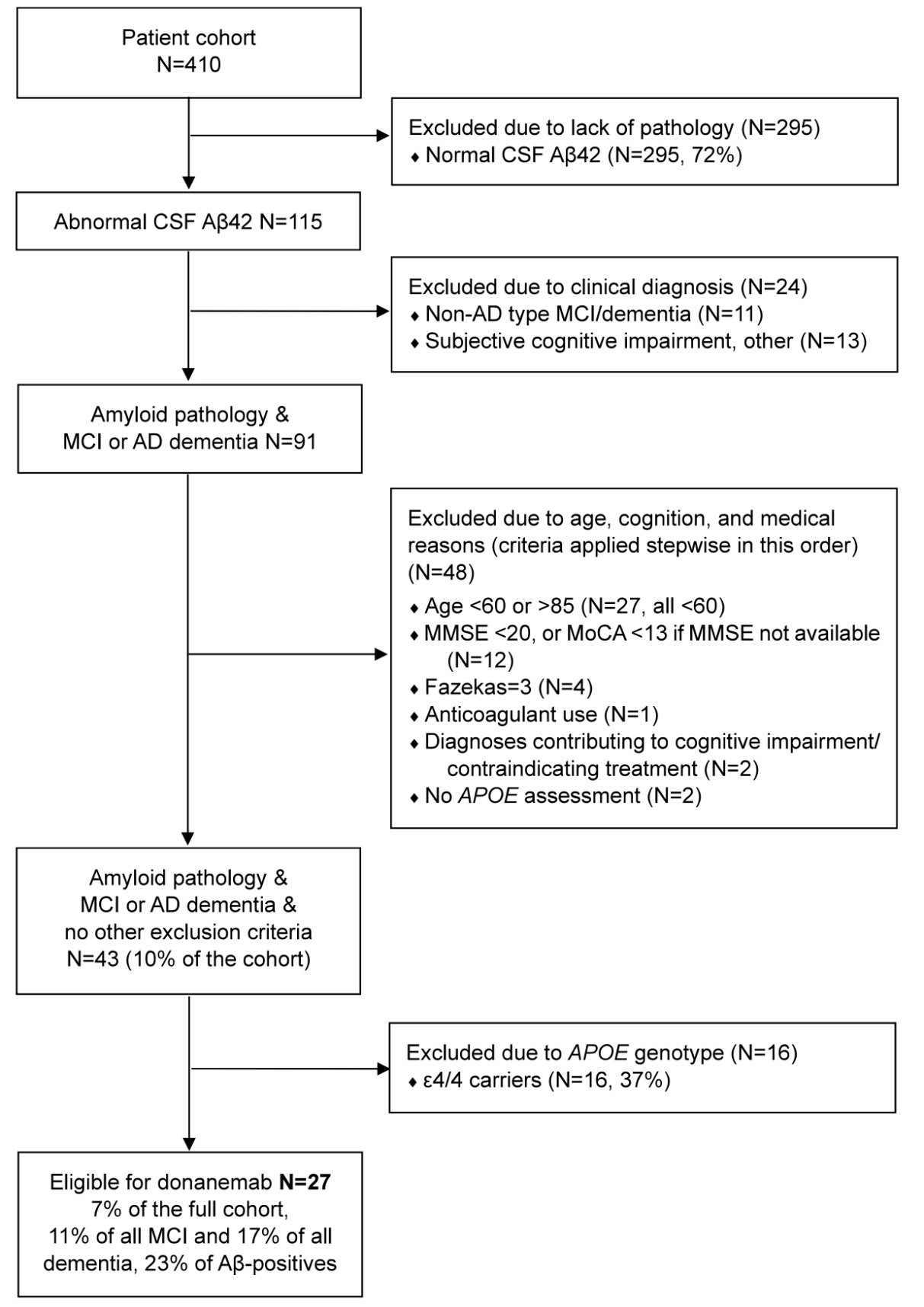


Supplementary Figure 2. Potential eligibility for donanemab treatment, using the data-driven cut-off for CSF Aβ42 to determine amyloid pathology

Eligibility was assessed according to the donanemab appropriate use recommendations (AUR) (Rabinovici et al., 2025). Abnormal CSF Aβ42 regardless of tau was considered sufficient evidence for AD pathology. Exclusion of *APOE* ε4/4 carriers (final step) is the EMA requirement for patients in the EU.
